# Coxsackievirus A16 Infection Induces Neural Cell and Non-Neural Cell Apoptosis *In Vitro*

**DOI:** 10.1371/journal.pone.0111174

**Published:** 2014-10-28

**Authors:** Zhaolong Li, Jinghua Yu, Li Liu, Zhenhong Wei, Elana S. Ehrlich, Guanchen Liu, Jingliang Li, Xin Liu, Hong Wang, Xiao-fang Yu, Wenyan Zhang

**Affiliations:** 1 Institute of Virology and AIDS Research, The First Hospital of Jilin University, Changchun, China; 2 Department of Pediatric Pulmonology, The First Hospital of Jilin University, Changchun, China; 3 College of Life Science, Jilin University, Changchun, China; 4 Department of Biological Sciences, Towson University, Towson, Maryland, United States of America; 5 Department of Molecular Microbiology and Immunology, Johns Hopkins Bloomberg School of Public Health, Baltimore, Maryland, United States of America; Indian Institute of Science, India

## Abstract

Coxsackievirus A16 (CA16) is one of the main causative pathogens of hand, foot and mouth disease (HFMD). Viral replication typically results in host cell apoptosis. Although CA16 infection has been reported to induce apoptosis in the human rhabdomyosarcoma (RD) cell line, it remains unclear whether CA16 induces apoptosis in diverse cell types, especially neural cells which have important clinical significance. In the current study, CA16 infection was found to induce similar apoptotic responses in both neural cells and non-neural cells *in vitro,* including nuclear fragmentation, DNA fragmentation and phosphatidylserine translocation. CA16 generally is not known to lead to serious neurological symptoms *in vivo*. In order to further clarify the correlation between clinical symptoms and cell apoptosis, two CA16 strains from patients with different clinical features were investigated. The results showed that both CA16 strains with or without neurological symptoms in infected patients led to neural and muscle cell apoptosis. Furthermore, mechanistic studies showed that CA16 infection induced apoptosis through the same mechanism in both neural and non-neural cells, namely via activation of both the mitochondrial (intrinsic) pathway-related caspase 9 protein and the Fas death receptor (extrinsic) pathway-related caspase 8 protein. Understanding the mechanisms by which CA16 infection induces apoptosis in both neural and non-neural cells will facilitate a better understanding of CA16 pathogenesis.

## Introduction

Hand, foot, and mouth disease (HFMD) is a febrile exanthematous disease mostly prevalent in children younger than 5 years of age. The symptoms of HFMD are vesicles on the palmar and plantar surfaces of the hands and feet, and on the buccal mucosa, tongue and buttocks [1], [2], [3]. Both coxsackievirus A16 (CA16) and enterovirus 71 (EV71) are the major pathogens responsible for HFMD, circulating alternatively or together in different years [4], [5], [6], [7], [8]. From 2001 to 2007, surveillance data in Singapore showed that the predominant circulating virus was CA16 in the 2002, 2005 and 2007 epidemics and EV71 in the 2006 epidemic [4]. The seroprevalence data in Guangdong Province, China showed that EV71 was responsible for 44.6%, 46.4% and 31.8% of reported cases, while CA16 accounted for 70.3%, 51.6% and 47.3% in 2007, 2008 and 2009, respectively. Of all tested individuals, 32.4% and 29.4% respectively exhibited neutralizing antibodies against both viruses in 2007 and 2008 [9].

As the main etiological agents of HFMD, CA16 and EV71 share some common structural characteristics and belong to the *Enterovirus* genus of the *Picornaviridae* family [10], [11], [12], [13], but they have different clinical features. EV71 infection usually causes severe symptoms, including encephalitis, aseptic meningitis, herpangina, myocarditis, acute flaccid paralysis, and pulmonary edema or hemorrhage [14], [15], and even leads to death of infected children. Studies on the molecular basis and mechanisms of the host response to viral infection determined that apoptosis induced by EV71, which has been observed in different cell lines including human glioblastoma, neuroblastoma, endothelial, rhabdomyosarcoma (RD) and African green monkey kidney (Vero) cells *in vitro*, may contribute to the pathogenesis of infection [16], [17], [18], [19], [20]. Accumulating evidence has demonstrated that CA16 infection also causes neurological complications and death [21], [22], [23]. However, the mechanism(s) responsible for CV16-induced pathogenesis remains unclear. Zhu et al. previously reported the induction of apoptosis by CA16 in non-neural cell lines [24], but whether this virus induces apoptosis in neural cell lines remains unknown.

Previous studies have shown that a number of neurotropic viruses can cause damage to the central nervous system through induction of apoptosis [25], [26]. One of two main mechanisms of apoptosis is the extrinsic pathway (i.e., Fas death receptor pathway), which activates caspase 8 and caspase 10 in response to external stimuli [27], [28], [29], [30], [31]. The other apoptotic mechanism involves the intrinsic pathway (i.e., mitochondrial pathway), which activates cleavage of pro-caspase 9 in response to internal stimuli [32]. Once initiator caspases 8 and 9 activate caspase 3, proteolytic cleavage of cellular target proteins and apoptosis will occur. Different caspases have been reported to be activated by different viruses. For example, EV71 may activate caspases 3, 8 and 9 [17], while poliovirus mainly activates caspases 3 and 9 [33].

In this study, we investigated whether CA16 infection can induce extensive apoptosis in different cell lines *in vitro*, especially in neural cell lines. We also investigated whether CA16-induced apoptosis occurs through different cell signaling pathways in different types of cell lines, as has been demonstrated for EV71. Clarifying the differences in pathogenic mechanisms between EV71 and CA16 is critical, and this study provides useful information for understanding the correlation between pathogenesis and clinical symptoms of CA16.

## Results

### CA16 induces cytopathic effects (CPEs) in diverse cell types

**Figure 1:**
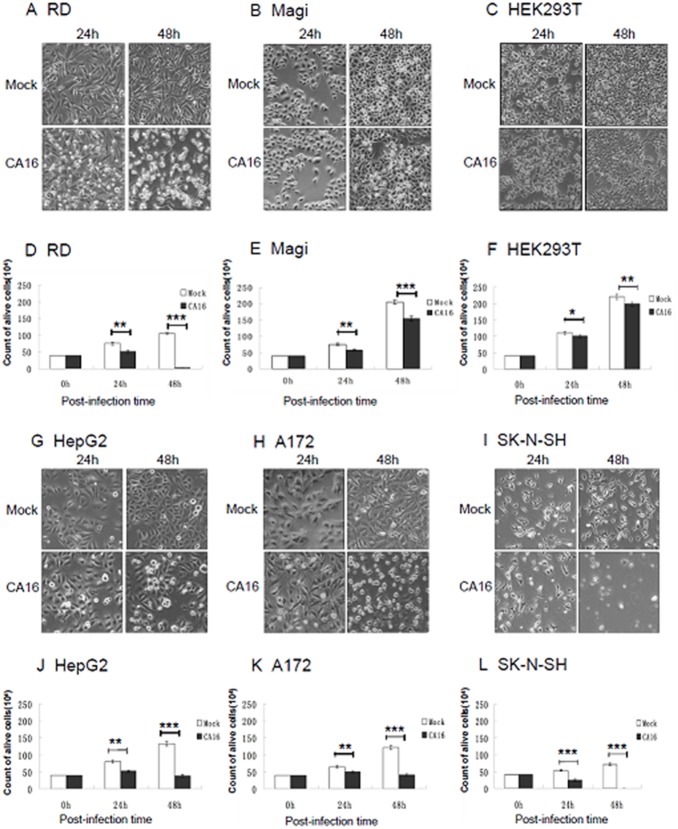
CA16 induces CPEs in both neural and non-neural cells. Different cell lines were inoculated with CA16 SHZH05 virus at the MOI of 0.2 or DMEM as a negative control. At 24 h and 48 h post-infection, cells were imaged via light microscopy, stained with Trypan Blue and counted. Representative images of (A) RD, (B) Magi, (C) HEK293T, (G) HepG2, (H) A172 and (I) SK-N-SH cell lines are shown of three individual experiments performed for each cell line. Counts of live (D) RD, (E) Magi, (F) HEK293T, (J) HepG2, (K) A172 and (L) SK-N-SH cells (n = 3) were statistically analyzed for differences between infected and control groups. *P<0.05, **P<0.01, ***P<0.001.

The diverse tissue tropism of CA16 in infected individuals led us to investigate the differences in its infectivity or virulence in diverse cell types. Here, we first examined the CPEs of the CA16 SHZH05 strain during infection of different human cell lines. RD (rhabdomyosarcoma), Magi (human cervical cancer), HEK293T (human embryonic kidney 293T), hepatocellular carcinoma (HepG2), glioblastoma (A172) and neuroblastoma (SK-N-SH) cells were inoculated with the CA16 SHZH05 strain at the multiplicity of infection (MOI) of 0.2 or Dulbecco’s Modified Eagle’s Medium (DMEM) as a negative control. Morphological changes associated with infection were examined at 24 h and 48 h post-infection. In the RD and SK-N-SH (Fig. 1A and 1I) lines, numerous cells appeared rounded and detached from the bottom of the culture dish at 24 h and 48 h post-infection, representing typical CPEs induced by CA16. In the HepG2 (Fig. 1G) and A172 (Fig. 1H) cell lines, clear CPEs were observed at 48 h post-infection, but no obvious CPEs were seen in Magi (Fig. 1B) and HEK293T (Fig. 1C) cells, even at 48 h post-infection. Mock-infected cells showed no obvious CPE and remained intact. These results demonstrated that CA16 selectively induced cell loss or damage in some cultured cells to various extents in different cell lines.

By using Trypan Blue exclusion, live cell numbers were counted to confirm the level of cell death induced by CA16 infection compared to control treatment. Infection with CA16 resulted in clearly decreased cell numbers at 24 h and 48 h post-infection in RD (Fig. 1D), HepG2 (Fig. 1J), A172 (Fig. 1K) and SK-N-SH (Fig. 1L) cells, especially at 48 h. Decreases in cell numbers also were noted for the Magi (Fig. 1E) and HEK293T (Fig. 1F) cell lines, although they displayed no obvious CPEs even at 48 h post-infection (Fig. 1B and 1C, respectively). These results suggested that the virus infection could decrease cell numbers by inhibiting cell growth but without necessarily inducing CPE.

### CA16 infection promotes nuclear fragmentation

**Figure 2:**
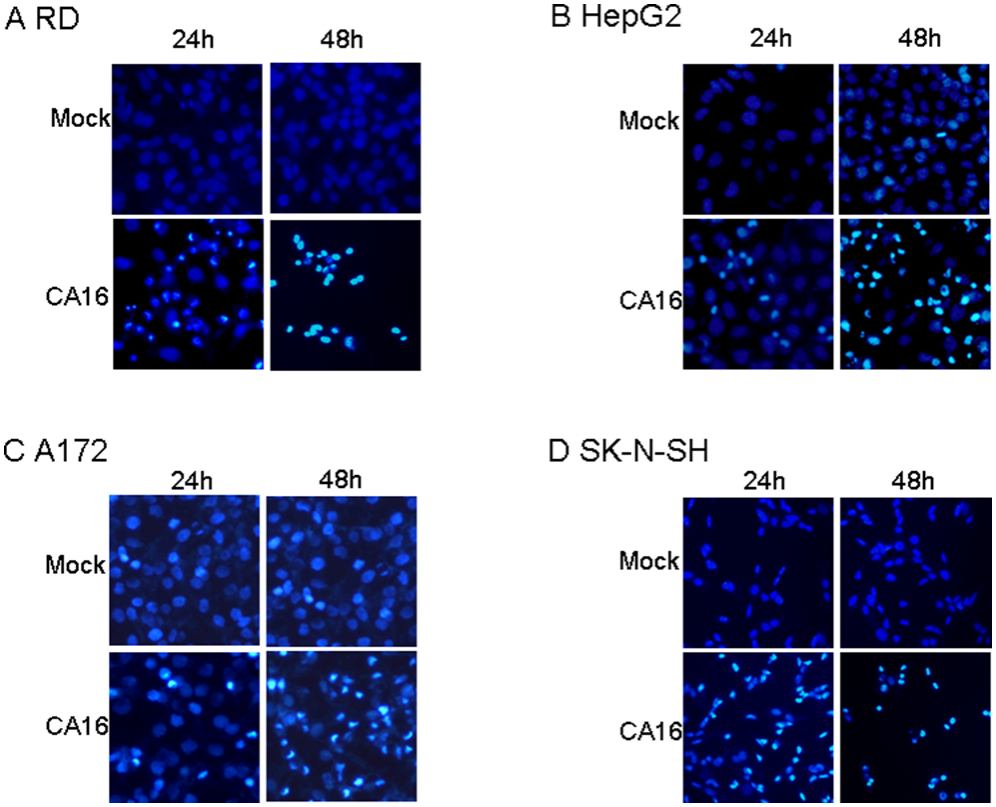
Nuclear fragmentation occurs in CA16-infected cells. (A) RD, (B) HepG2, (C) A172 and (D) SK-N-SH cell lines were inoculated with CA16 SHZH05 virus at the MOI of 0.2 or DMEM as a negative control. After 24 h or 48 h of infection, cells were stained with Hoechest 33258. Nuclear fragmentation was observed via fluorescence microscopy. Representative images are shown of three individual experiments (n = 3) performed for each cell line.

Although CA16 does not usually lead to nervous symptoms, its potential to cause cell damage in neural cell lines was evaluated by staining with the nuclear fluorescent dye Hoechst 33258, which is taken up in apoptotic cells. Highly condensed chromosomes are stained bright blue, whereas uncondensed chromosomes are stained weakly. At 48 h after infection with the CA16 SHZH05 strain at the MOI of 0.2, increased nuclear fluorescence was observed in neural cell lines A172 (Fig. 2C) and SK-N-SH (Fig. 2D) or non-neural cell lines RD (Fig. 2A) and HepG2 (Fig. 2B). Therefore, CA16 infection may lead to cell damage through apoptosis in both neural and non-neural cell lines. Meanwhile, condensed chromatin first appeared at 24 h after infection in RD (Fig. 2A) and SK-N-SH (Fig. 2D) cells, which was earlier than that in A172 (Fig. 2C) and HepG2 (Fig. 2B) cells. These results further demonstrated the different sensitivities of different lines, whether neural or non-neural cells, to the CA16 virus.

### CA16 infection induces DNA fragmentation

**Figure 3:**
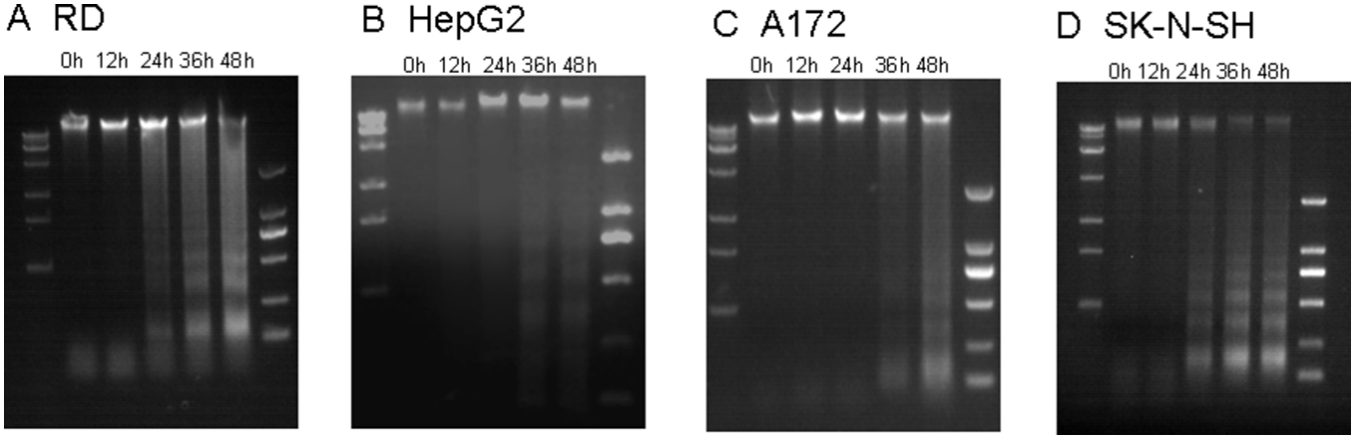
CA16 infection induces DNA fragmentation. (A) RD, (B) HepG2, (C) A172 and (D) SK-NSH cell lines were infected with CA16 SHZH05 virus at the MOI of 0.2, 0.2, 0.2 and 0.05, respectively, and collected at 0 h, 12 h, 24 h, 36 h or 48 h as indicated. DNA was extracted from cells and analyzed for fragmentation via agarose gel electrophoresis. Representative images are shown of three individual experiments (n = 3) performed for each cell line.

DNA fragmentation is a key feature of apoptosis, which involves the activation of endogenous endonucleases and subsequent cleavage of chromatin into internucleosomal fragments with lengths of multiples of 180 base pairs (bp). In this study, apoptotic DNA fragmentation was analyzed using gel electrophoresis following CA16 SHZH05 infection in each cell line. DNA fragmentation was observed in RD cells (MOI of 0.2, Fig. 3A) and SK-N-SH cells (MOI of 0.05, Fig. 3D) beginning at 24 h post-infection. The SK-N-SH cells were inoculated initially with virus at a low MOI in order to obtain a sufficient number of cells for testing. DNA fragmentation was not observed in HepG2 (MOI of 0.2, Fig. 3B) and A172 (MOI of 0.2, Fig. 3C) cells until 36 h after CA16 infection, further demonstrating the sensitivities of different cell lines to CA16-induced apoptosis.

### Confirmation of CA16-induced cell apoptosis by Annexin V and propidium iodide (PI) staining

**Figure 4:**
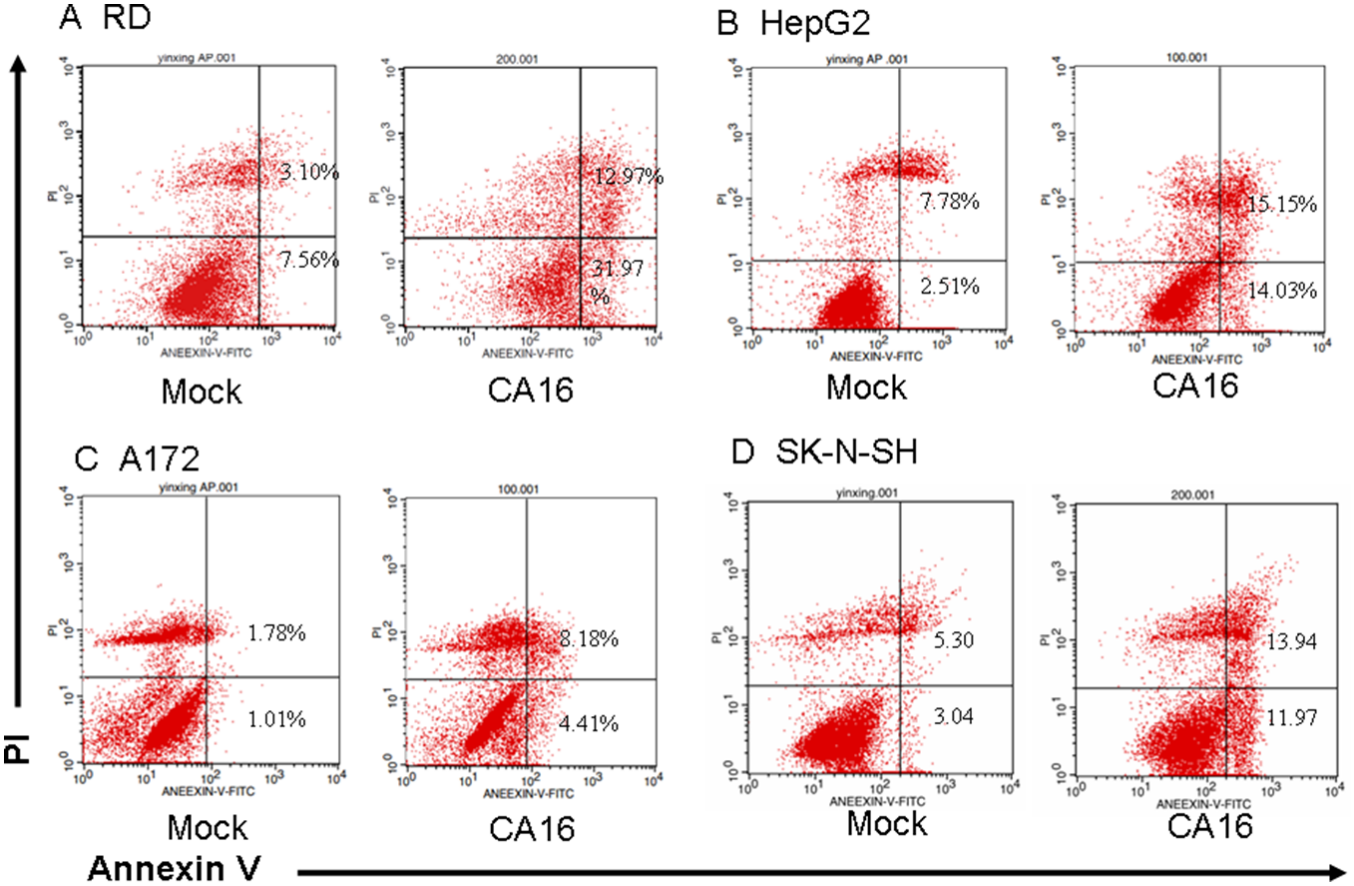
Membrane changes associated with apoptosis in CA16-infected cells. (A) RD, (B) HepG2, (C) A172 and (D) SK-N-SH cells were inoculated with CA16 SHZH05 virus at the MOI of 1.0, 2.0, 1.0 and 1.0, respectively, or DMEM as a negative control for 24 h. Cells were washed with PBS, incubated with FITC-labeled Annexin V and stained with PI, followed by analysis via flow cytometry. Annexin V-positive/PI-negative cells were considered to be in early phase apoptosis. Annexin V-positive/PI-positive cells were considered to be in late phase apoptosis. Representative images are shown of three individual experiments (n = 3) performed for each cell line. *P<0.05.

Early in apoptosis, phosphatidylserine is translocated to the outside of the plasma membrane. To further provide evidence for CA16-induced apoptosis, we used Annexin V and PI staining to analyze phosphatidylserine translocation. In order to induce extensive early apoptosis, different amounts of CA16 SHZH05 virus were used to infect different cell lines. RD, HepG2, A172 and SK-N-SH cells were inoculated with CA16 for 24 h at the MOI of 1.0, 2.0, 1.0 and 1.0, respectively, or with DMEM as a negative control, followed by Annexin V and PI staining. Early apoptotic cells will exclude PI and stain with Annexin V, while late stage apoptotic cells and necrotic cells will stain with PI and Annexin V due to the passage of these dyes into the nucleus where they bind to DNA. At 24 h post-infection, the proportion of early apoptotic cells (lower right) increased from 7.56% to 31.97% in the RD cell line (Fig. 4A), 2.51% to 14.03% in the HepG2 cell line (Fig. 4B), 1.01% to 4.41% in the A172 cell line (Fig. 4C), and 3.04% to 11.97% in the SK-N-SH cell line (Fig. 4D). The proportion of late apoptotic cells (top right) increased from 3.10% to 12.97% in the RD cell line (Fig. 4A), 7.78% to 15.15% in the HepG2 cell line (Fig. 4B), 1.78% to 8.18% in the A172 cell line (Fig. 4C), and 5.30% to 13.94% in the SK-N-SH cell line (Fig. 4D). These results further supported that CA16 infection induces apoptosis in RD, HepG2, A172 and SK-N-SH cells.

### Activation of caspases by CA16 infection

**Figure 5:**
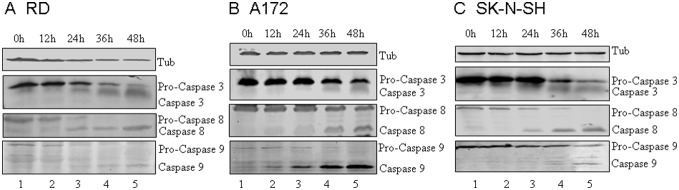
CA16 infection results in pro-caspase cleavage in neural and non-neural cell lines. Neural and non-neural cell lines were infected with CA16 SHZH05 virus at the MOI of 0.2 and harvested at 0 h, 12 h, 24 h, 36 h and 48 h. The cells were lysed and analyzed via immunoblotting against caspases 3, 8 and 9 and tubulin where indicated. (A) RD cells, (B) A172 cells, (C) SK-N-SH cells.

Caspases are a family of cysteine proteases involved in regulating apoptosis [28]. Apoptosis is mediated by caspase 3, which cleaves a number of cellular proteins to trigger apoptosis. Caspase 3 is activated through proteolytic cleavage by upstream caspases such as caspase 8 and caspase 9, which are activated in response to proapoptotic stimuli [27], [28], [29], [30], [31], [32]. EV71 infection was previously reported to induce apoptosis through different apoptotic pathways in non-neural cells and neural cells [16], [17]. In our study, we investigated the activation of caspase proteins by observing pro-caspase cleavage, and RD, A172 and SK-N-SH cells were chosen to assess the differences in activation of different apoptotic pathways by EV71 and CA16. Activation of caspases 3, 8 and 9 was observed in RD cells at 24 h post-infection (Fig. 5A). In A172 cells, activation of caspase 8 and caspase 9 was noted at 12 h post-infection (Fig. 5B), and caspase 3 at 36 h post-infection. In SK-N-SH cells, caspases 3, 8 and 9 were activated at 24 h post-infection (Fig. 5C). The timing of caspase 3 activation was consistent with our observations of morphological changes and DNA fragmentation. The differences in caspase 3 activation by upstream caspase 8 and caspase 9 may be due to the unique timing of cell signal transduction in each cell line. These results suggest that CA16 infection activates caspases 3, 8 and 9 in both neural and non-neural cells (Fig. 5).

**Figure 6:**
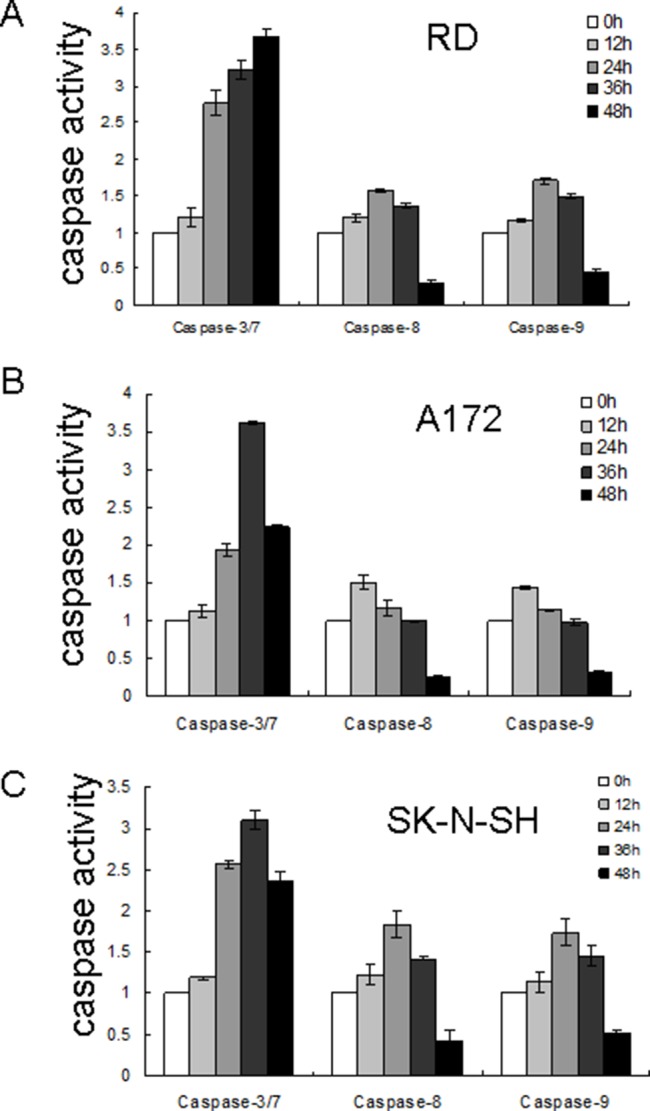
CA16 infection induces caspase activity in neural and non-neural cell lines. Neural and non-neural cell lines were infected with CA16 SHZH05 virus at the MOI of 0.2 and harvested at 12 h, 24 h, 36 h and 48 h. Activities of caspase 3/7, caspase 8 and caspase 9 were quantified and compared to the 0 h time point. (A) RD cells, (B) A172 cells, (C) SK-N-SH cells. Results are shown as the combined data set from three repeat experiments and presented as the mean ± SD.

In addition to observing pro-caspase cleavage, we also examined caspase activity in neural and non-neural cells using a caspase activity assay (Fig. 6). Increases in the enzymatic activities of caspase 3/7, caspase 8 and caspase 9 were observed in CA16-infected cells compared to controls. Caspase 3/7 activity was increased to a higher level than those of caspase 8 and caspase 9. After CA16 infection, caspase 8 and caspase 9 activities first increased and then decreased from 36 h post-infection in RD and SK-N-SH cells or from 24 h post-infection in A172 cells. Caspase 3 activity increased in a time-dependent manner until 48 h in RD cells. However, in A172 and SK-N-SH cells, caspase 3 activity peaked at 36 h and then decreased at 48 h post-infection. Therefore, patterns of caspase activity differed in the various cell types examined.

### Apoptosis is induced by circulating CA16 strains with different clinical features

In a previous study, we isolated and characterized several CA16 strains with different clinical features [34], [35]. Here, we used virus strains CC045 and CC097 isolated from patients with different clinical symptoms to evaluate the effects of CA16 on cell apoptosis. Clinical features of the patient infected with CC045 were skin rash, herpangina, vomiting and myoclonic jerk; meanwhile, those of the patient infected with CC097 were skin rash, herpangina and fever, as well as severe complications of pneumonia (Table 1).

**Table 1:** Clinical features of CC045 and CC097 strains.

	CC045	CC097
Gender	Female	Male
Age	3 Y	1 Y
Symptoms	Skin rash	Skin rash
	Herpangina	Herpangina
	Vomiting	Fever
	Myoclonic jerk	
Complications	No	Bronchial
		pneumonia

**Figure 7:**
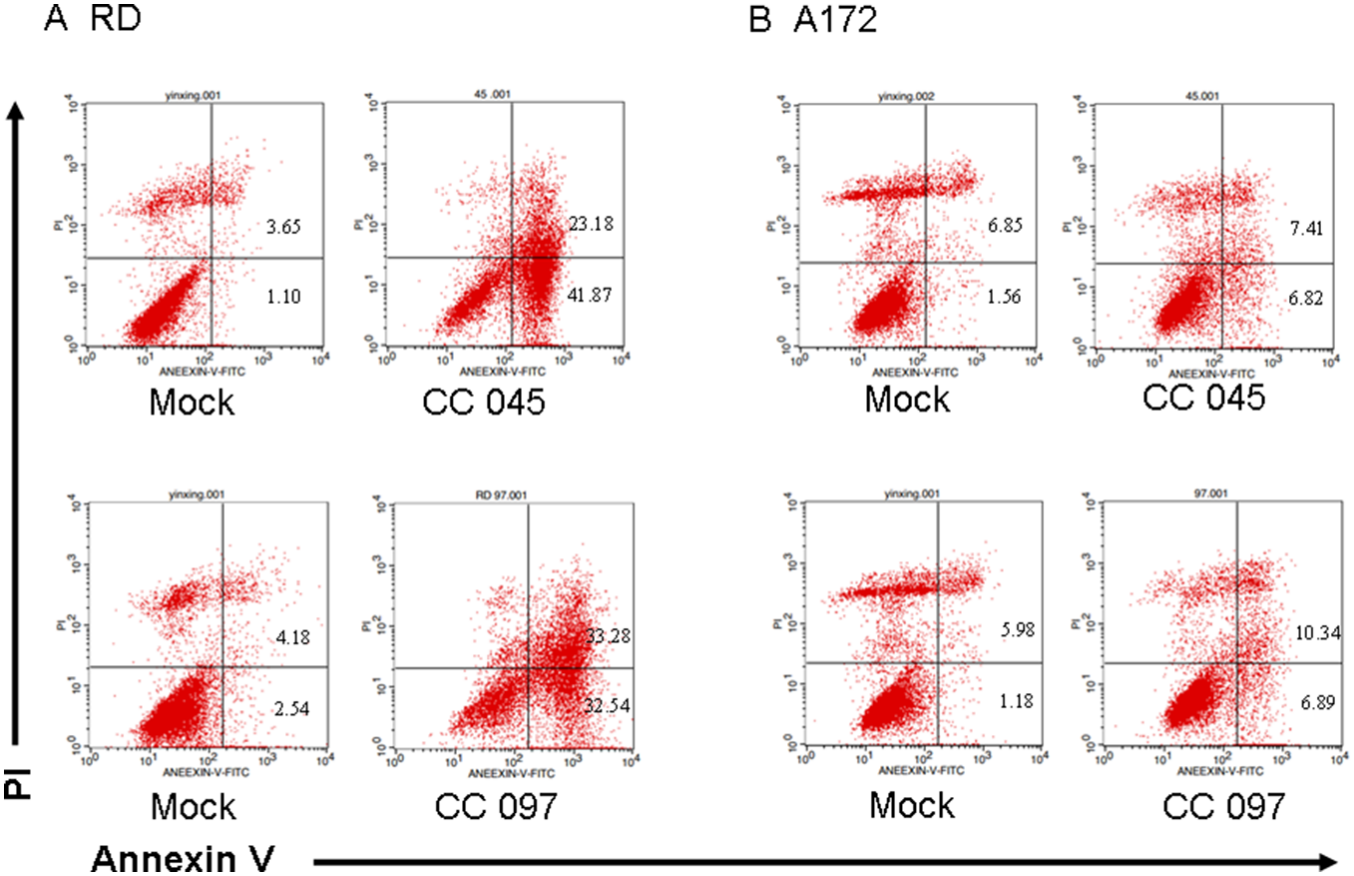
Circulating CA16 strains CC045 and CC097 induce apoptosis. (A) RD cells were inoculated with CC045 and CC097 viruses at the MOI of 1.0 or DMEM as a negative control for 24 h. Cells were washed with PBS and incubated with a FITC-labeled Annexin V and stained with PI, followed by analysis via flow cytometry. Annexin V-positive/PI-negative cells were considered to be in early phase apoptosis. Annexin V-positive/PI-positive cells were considered to be in late phase apoptosis. (B) A172 cells were inoculated with CC045 or CC097 at the MOI of 1.0 for 24 h or DMEM as a negative control for Annexin-PI staining. Representative images are shown of three individual experiments (n = 3) performed for each cell line. *P<0.05.

In these experiments, CC045 was used at the MOI of 1.0 to induce extensive apoptosis in neural and non-neural cell lines. In the non-neural RD cell line at 24 h post-infection, the proportion of early apoptotic cells (lower right) increased from 1.10% to 41.87%, and that of late apoptotic cells (top right) increased from 3.65% to 23.18% (Fig. 7A). In the neural A172 cell line at 24 h post-infection, the proportion of early apoptotic cells (lower right) increased from 1.56% to 6.82%, and that of the late apoptotic cells (top right) increased from 6.85% to 7.41% (Fig. 7B).

The other virus patient virus CC097 also was used to infect cells at the MOI of 1.0. In the non-neural RD cell line at 24 h post-infection, the proportion of early apoptotic cells (lower right) increased from 2.54% to 32.54%, and that of the late apoptotic cells (top right) increased from 4.18% to 33.28% (Fig. 7A). Meanwhile, in the neural A172 cell line at 24 h post-infection, the proportion of early apoptotic cells (lower right) increased from 1.18% to 6.89%, and that of the late apoptotic cells (top right) increased from 5.98% to 10.34% (Fig. 7B). These results suggest that these circulating CA16 strains associated with different clinical features could induce apoptosis at the same level in muscle and neural cells. This finding is in agreement with our data showing that CA16 SHZH05 has the same ability to induce apoptosis as circulating CA16 strains.

### The correlation between extracellular replication kinetics of CA16 and the degree of apoptosis induced in diverse cell lines

**Figure 8:**
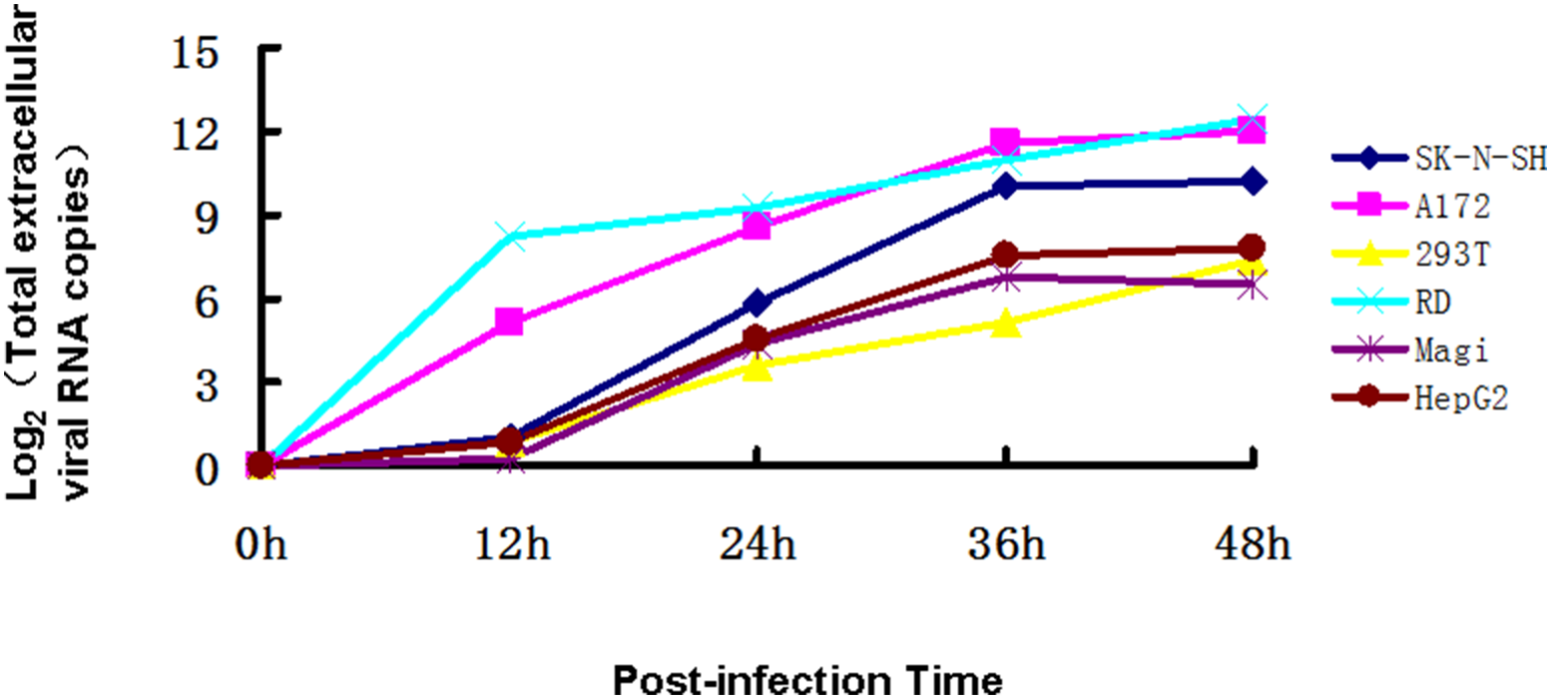
Replication kinetics of CA16 in diverse cell lines. RD, Magi, HEK293T, HepG2, A172 and SK-N-SH cell lines were infected with CA16 SHZH05 virus at the MOI of 0.2 for 1 h, and viral supernatants were collected at various time points post-infection. Extracellular virus RNA copy numbers were determined by RT-qPCR at 0 h, 12 h, 24 h, 36 h and 48 h. The CA16 RNA copy number at 1 h was set as zero. Each point represents the mean ± SD of three separate experiments performed.

To further examine the relationship between the induction of apoptosis and the replication kinetics of CA16 viruses in diverse cell types, we established growth curves for CA16 in different cell lines. The cells were infected with CA16 SHZH05 at the MOI of 0.2. Virus adsorption was performed at 37°C for 1 h to permit virus binding and entry into host cells. The extracellular viral RNA copy number (Fig. 8) was determined by RT-qPCR at each indicated time point. As shown in Fig. 8, little viral RNA was detected in the Magi, HEK293T, HepG2 and SK-N-SH cell lines, while much higher levels of viral RNA were detected in the RD and A172 cell lines at 12 h post-infection. Thereafter, amounts of total extracellular viral RNA increased between 12 h to 36 h in all cell lines and then remained almost unchanged at 48 h post infection. The total extracellular viral RNA in the RD, A172 and SK-N-SH cell lines maintained higher levels at 48 h compared to those in the HEK293T, HepG2 and Magi cell lines. Thus, the replication kinetics of CA16 in different cell lines relatively correlates with the observed extent of apoptosis in the tested cell lines.

## Discussion

CA16 infection generally does not lead to nervous symptoms in the clinic, but here we found that CA16 infection induced apoptosis not only in non-neural cells but also in neural cells *in* *vitro*. Increasing evidence shows that CA16 is also associated with damage to muscle and brain tissue [12], [13], [36], [37], [38]. In this study, the human rhabdomyosarcoma RD cell line was chosen to evaluate the tropism and damage to muscle, and the human A172 glioblastoma cell line, was chosen to assess the tropism and damage to the brain. Damage to muscle and brain was previously reported in neonatal mice [39]. Human neuroblastoma SK-N-SH cells, human embryonic kidney 293T cells and human hepatocellular carcinoma HepG2 cells were also utilized in our study. The apoptotic response to CA16 infection was examined by evaluating standard markers of apoptosis, including CPEs, chromatin condensation, nuclear fragmentation, DNA fragmentation and phosphatidylserine translocation. Evidence of all markers of apoptosis in RD, HepG2, A172 and SK-N-SH cells indicated that CA16 infection induced extensive apoptosis in different cell types including neural cell lines, even though CA16 infection does not lead to nervous symptoms in patients. To further clarify the correlation between the clinical symptoms and neural cell apoptosis, circulating CA16 viruses CC045 and CC097 isolated from patients with different clinical symptoms, in addition to the non-circulating CA16 SHZH05 strain, were chosen for evaluation of apoptosis *in* *vitro*. Although CC045 and CC097 strains were obtained from patients with varying clinical symptoms, both were able to induce apoptosis *in* *vitro*. While this observation does not explain why CA16 is generally associated with mild and benign central nervous symptoms, it does account for the small number of severe cases that resulted in central nervous system damage [38], [40], [41]. These observations are consistent with reports of CA16 infection in neonatal mice resulting in pathological changes in skeletal muscle and brain. Although it remains unclear why EV71 infections more often result in a potentially fatal outcome than CA16 infections, this study suggests that in addition to apoptosis, processes such as inflammation and cell tropism may be responsible for the pathogenic differences observed between these viruses.

To determine whether the observed apoptosis was dependent on viral replication, we analyzed the viral growth kinetics of CA16 in different cell lines. We found that at 48 h post-infection of CA16, viral replication in RD, and A172 SK-N-SH cells was substantially different from that in Magi, HepG2 and HEK293T cell lines. Replication kinetics of CA16 in the RD, SK-N-SH and A172 cell lines were basically consistent with observations of CPEs (Fig. 1) although the extracellular viral RNA level in SK-N-SH was lower than that in the RD and A172 cell lines, which was due to severe CPEs. In the Magi and HEK293T cell lines, extracellular viral RNA increased over time, although we did not observe obvious CPEs (Fig. 1), implying that the viruses were externalized from cells through a mechanism without causing cell damage. A medium level of extracellular viral RNA was observed in the HepG2 cell line which had CPE. The above results suggest that CA16-induced apoptosis is not completely related to viral replication, and the detailed mechanism needs to be further investigated.

Caspases are a family of cysteine proteases involved in regulating cytokine maturation and apoptosis [42]. They have been demonstrated to be activated by poliovirus, a small non-enveloped RNA virus with a genome similar to that of CA16 [33]. Specifically, caspase 9 and caspase 3 were shown to be essential for the development of poliovirus-induced apoptosis [43], [44]. The activation of caspase 8, in addition to caspase 3 and caspase 9, was investigated in response to infection with EV71. The mitochondrial pathway-specific caspase 9 was found to be activated in both neural and non-neural cells, but the death receptor pathway-associated caspase 8 was activated only in non-neural cells [17].

In order to investigate the pathways responsible for CA16-associated apoptosis in different cell types in this study, experiments were performed in neural A172 cells, SK-N-SH cells and non-neural RD cells. We found that A172, SK-N-SH and RD cells shared the same apoptotic pathways, with caspase 3, caspase 8 and caspase 9 all being activated after CA16 infection. Using a caspase activity assay, we found that in the non-neural RD cell line, caspase 3 was persistently activated. However, in the neural A172 and SK-N-SH cell lines, caspase 3 activities increased until 36 h, followed by a decrease in activity. It is possible that neural cells may have a protective mechanism to avoid long-term damage to the nervous system by down-regulating caspase 3 activity following CA16 infection.

In conclusion, we determined that CA16 could induce apoptosis in neural and non-neural cells. The apoptotic pathways induced by CA16 infection were similar. This study will help further understanding of the pathogenic mechanism(s) of CA16, especially differences in clinical manifestations. This research also may aid the development of novel anti-viral drugs and help to prevent neurological complications associated with HFMD.

## Materials and Methods

### Ethics Statement

This study has obtained ethics approval from the ethics committee at the First Hospital of Jilin University. Written informed consent was obtained from the parents of all the children involved in our study.

### Cells and viruses

Human rhabdomyosarcoma RD cells (no. CCL-136), human hepatocellular carcinoma HepG2 (no. HB-8065), human neuroblastoma SK-N-SH (no. HTB-11), human glioblastoma A172 (no. CRL-1620) and human embryonic kidney 293T HEK293T (no. CRL-11268) cells were obtained from American Type Culture Collection (Manassas, VA, USA). Human cervical cancer Magi cells (no. 3522) were obtained from the AIDS Research and Reference Reagents Program, Division of AIDS, NIAID gifted by Julie Overbaugh [45]. Cells were cultured in Dulbecco’s modified Eagle’s medium (Hyclone, Logan, UT, USA) supplemented with 10% heat-inactivated (56°C, 30 min) fetal calf serum (GIBCO BRL, Grand Island, NY, USA) and maintained at 37°C with 5% CO_2_ in a humidified atmosphere. The CA16 SHZH05 virus (GenBank accession no. EU262658) used in most experiments in this study was a gift from Qi Jin at the Institute of Pathogen Biology (Beijing, China). CA16 virus strains Changchun045 (CC045, GenBank accession no. KF055241) and Changchun097 (CC097, GenBank accession no. KF055244) were isolated from patients at the First Hospital of Jilin University and characterized by our laboratory [34].

### Isolation of viruses from HFMD patients and viral characterization

Vero cells were used to isolate CA16 virus strains of CC045 and CC097 from the throat swabs of patients with HFMD in 2010. The viral samples were diluted in DMEM medium and sterilized by passing through 0.22-µm filters (Millipore, Bedford, MA, USA); 300 µL of each filtered sample was inoculated into T25 flasks containing approximately 50% confluent Vero cells. The CPEs of the infected cultures were monitored daily. Culture supernatants from infected Vero cells with CPEs were collected, aliquoted, viral-titered and stored at −80°C. Viral titers were determined according to the Reed–Muench method (Reed and Muench, 1938).

### Virus preparation

CA16 SHZH05, CC045 and CC097 viruses were propagated using Vero (African green monkey kidney) cells, as previously described [46]. Briefly, cells were grown to 80% confluence in a T75 flask, washed twice with phosphate-buffered saline (PBS) and incubated with virus at 37°C for 1 h. During adsorption, the flask was gently agitated at 15 min intervals. Following adsorption, the virus-containing medium was replaced with fresh medium containing 2% FBS, followed by incubation at 37°C in 5% CO_2_. Once 90% of the cells showed CPEs, the viral supernatant was harvested and centrifuged at 4,000 rpm for 5 min. The clear supernatant was then transferred to a new tube and stored at −80°C.

### Virus titer

The virus titer was determined by measuring the 50% tissue culture infective dose (TCID_50_) in a microtitration assay, as described previously [47]. Briefly, Vero cells were seeded into 96-well plates and incubated at 37°C for 24 h. Virus-containing supernatant was serially diluted 10 fold, and 100 µL was added per well in triplicate. CPEs were observed once per day until the experimental endpoint. The viral titer was determined in Vero cells according to the Reed-Muench method (Reed and Muench, 1938).

### CPE observation

For observing CPEs, cells were grown on a culture dish and infected by CA16 at the MOI of 0.2 at indicated time points. Morphological changes were observed and photographed under a light microscope (Olympus IX51, Tokyo, Japan).

### Cell counting using a hemocytometer

Trypan Blue (Sigma, St. Louis, MO, USA) was used as a vital stain. Live cells appeared colorless and bright (refractile) under phase contrast, while dead cells stained blue and were non-refractile. After staining with Trypan Blue at the final concentration of 0.2%, live cells were visualized and counted using a hemocytometer.

### Immunofluorescence assay and Hoechest 33258 staining

The nuclear stain Hoechest 33258 (Sigma) was used to visualize nuclear changes by fluorescence microscopy. Briefly, RD, HepG2, A172, and SK-N-SH cells were plated in 6-well plastic culture dishes (4×10^5^ cells/well) and infected by CA16 at the MOI of 0.2 for the indicated time periods. Thereafter, the cells were washed with PBS, fixed in 3.7% formaldehyde for 1 h, washed again with PBS and then stained with 5 mg/L Hoechst 33258 for 30 min. Nuclear changes were observed by fluorescence microscopy (Olympus IX51) at the excitation wavelength 350 nm with emission filter 460 nm.

### Annexin V-PI staining

Phosphatidylserine was detected by using the Annexin V-PI Apoptosis Detection Kit (BD, Franklin Lakes, NJ, USA) according to the manufacturer’s instructions. Cells were trypsinized, washed twice with cold PBS and resuspended in 200 µL 1X binding buffer. One hundred microliters of cell suspension was transferred to a 5 mL culture tube and incubated with 5 µL of FITC-Annexin V and 10 µL of PI (10 µg/mL) for 15 min at room temperature in the dark. Five hundred microliters of 1X binding buffer was added to each tube, and the cells were analyzed by flow cytometry according to the protocol of the Annexin V-PI Apoptosis Detection Kit.

### Determination of DNA fragmentation by agarose gel electrophoresis

Cells were trypsinized, and both adherent and floating cells were collected by centrifugation at 1,000×*g* for 5 min. The cell pellet was suspended in lysis buffer [Tris-HCl 10 mM, pH 7.4; edetic acid (EDTA) 10 mM, pH 8.0; Triton-100 0.5%] and incubated at 4°C for 30 min. The lysate was centrifuged at 25,000×*g* for 20 min. The supernatant was incubated with 20 g/L RNase A (2 µL) at 37°C for 1 h, then incubated with 20 g/L proteinase K (2 µL) at 37°C for 1 h. The supernatant was mixed with 5 M NaCl (20 µL) and isopropanol (120 µL), incubated at –20°C overnight and then centrifuged at 25,000×*g* for 15 min. After removing the supernatant, the DNA pellet was dissolved in TE buffer (Tris-HCl 10 mM, pH 7.4, EDTA 1 mM, pH 8.0) and separated by 2% agarose gel electrophoresis at 100 V for 50 min.

### Caspase activity assay

Caspase activity was analyzed using the caspase-Glo 3/7 Assay, caspase-Glo 8 Assay and caspase-Glo 9 (Promega, Madison, WI, USA) according to the manufacturer’s instructions. Briefly, 1×10^4^ cells (treated with or without CA16 virus at the MOI of 0.2) were collected at 0, 12, 24, 36 or 48 h as indicated and lysed using the manufacturer-provided homogeneous caspase 3/7 or caspase 8 reagent. The lysates were incubated at room temperature for 1.5 h before reading in a fluorometer at 485/530 nm. The relative caspase activity was calculated as the fold-changes of samples at 12, 24, 36 and 48 h (compared with sample at 0 h).

### Western blotting

Briefly, cell lysates were harvested and boiled in 1X loading buffer (0.08 M Tris, pH 6.8, with 2.0% SDS, 10% glycerol, 0.1 M dithiothreitol and 0.2% bromophenol blue) followed by separation on a 12% polyacrylamide gel. Proteins were transferred onto PVDF membranes for Western blot analysis. Antibodies against caspase 3, 8 or 9 (no. 9665, no. 9647 and no. 9508; Cell Signaling, Beverly, MA, USA) or mouse anti-tubulin (no. ab11323, Abcam, Cambridge, MA, USA) were diluted 1∶2000 in PBS plus 1% milk, followed by a corresponding AP-conjugated secondary antibody diluted 1∶1000. Proteins were visualized using the substrates nitroblue tetrazolium (NBT) and 5-bromo-4-chloro- 3-indolyl phosphate (BCIP) obtained from Sigma.

### RT-qPCR

Reverse transcription was carried out in a 20 µL volume containing 5 µL of RNA extracted from samples or from 10-fold serially diluted virus RNA standard (from 10 to 10^5^ copies) using a PrimeScript RT Kit (Takara, Japan) according to the manufacturer's instructions. The quantitative real-time polymerase chain reaction (qPCR) was carried out on an Mx3005P instrument (Agilent Technologies, Stratagene, USA) using the RealMaster Mix (SYBR Green) Kit (Takara) and primers designed using the VP1 conserved region sequences of CA16 as follows: CA16-F1, CATGCAGCGCTTGTGCTT; CA16-F2, CATGCAACGACTGTGCTTTC; CA16-R1, CACACAATTCCCCCGTCTTAC; CA16-R2, CATAATTCGCCCGTTTTGCT. The qPCR assay was carried out in a 20 µL volume consisting of 9 µL of 2.5× RealMaster Mix/20× SYBR Green solution containing HotMaster Taq DNA Polymerase, 1 µL of 5 µmol/L of each oligonucleotide primer and 4 µL of cDNA template. The target fragment amplification was carried out as follows: initial activation of HotMaster Taq DNA Polymerase at 95°C for 2 min, followed by 45 cycles of 95°C for 15 s, 57°C for 15 s and 68°C for 20 s.

### Statistical analysis

All data represent at least three independent experiments and are expressed as the mean ± standard deviation (SD). Statistical comparisons between two groups were made using a Student’s *t*-test, whereas comparisons between multiple groups were carried out using one-way ANOVA. *P*-values of less than 0.05 were considered to represent a statistically significant difference.
